# Human mesenchymal stromal cells and derived extracellular vesicles: Translational strategies to increase their proangiogenic potential for the treatment of cardiovascular disease

**DOI:** 10.1002/sctm.19-0432

**Published:** 2020-08-05

**Authors:** Timo Z. Nazari‐Shafti, Sebastian Neuber, Ana Garcia Duran, Zhiyi Xu, Eleftherios Beltsios, Martina Seifert, Volkmar Falk, Christof Stamm

**Affiliations:** ^1^ Department of Cardiothoracic and Vascular Surgery German Heart Center Berlin Berlin Germany; ^2^ German Centre for Cardiovascular Research, Partner Site Berlin Berlin Germany; ^3^ Berlin Institute of Health Center for Regenerative Therapies Charité – Universitätsmedizin Berlin Berlin Germany; ^4^ Berlin‐Brandenburg School for Regenerative Therapies Charité – Universitätsmedizin Berlin Berlin Germany; ^5^ Institute of Medical Immunology Charité – Universitätsmedizin Berlin, Corporate Member of Freie Universität Berlin, Humboldt‐ Universität zu Berlin, and Berlin Institute of Health Berlin Germany; ^6^ Division of Cardiovascular Surgery University of Zurich Zurich Switzerland

**Keywords:** angiogenesis, cardiovascular disease, extracellular vesicles, mesenchymal stromal cells, treatment

## Abstract

Mesenchymal stromal cells (MSCs) offer great potential for the treatment of cardiovascular diseases (CVDs) such as myocardial infarction and heart failure. Studies have revealed that the efficacy of MSCs is mainly attributed to their capacity to secrete numerous trophic factors that promote angiogenesis, inhibit apoptosis, and modulate the immune response. There is growing evidence that MSC‐derived extracellular vesicles (EVs) containing a cargo of lipids, proteins, metabolites, and RNAs play a key role in this paracrine mechanism. In particular, encapsulated microRNAs have been identified as important positive regulators of angiogenesis in pathological settings of insufficient blood supply to the heart, thus opening a new path for the treatment of CVD. In the present review, we discuss the current knowledge related to the proangiogenic potential of MSCs and MSC‐derived EVs as well as methods to enhance their biological activities for improved cardiac tissue repair. Increasing our understanding of mechanisms supporting angiogenesis will help optimize future approaches to CVD intervention.

## Graphical abstract

Mesenchymal stromal cells (MSCs) offer tremendous potential for the treatment of cardiovascular disease. Their predominant mode‐of action is the paracrine secretion of trophic factors and extracellular vesicles (EVs) that promote angiogenesis, inhibit apoptosis, and modulate immune responses. In this review, we discuss different strategies to enhance the angiogenic potential of MSCs and MSC‐derived EVs for improved cardiac tissue repair.

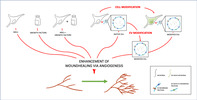

**Significance statement**

Mesenchymal stromal cells (MSCs) are currently being evaluated in clinical trials for the treatment of numerous diseases. Their therapeutic potential is mainly due to the factors they secrete. Studies have demonstrated that MSCs also produce extracellular vesicles that carry proteins, metabolites, lipids, and various RNAs. Based on their multifunctional properties, extracelullar vesicles are of great importance and interest in the development of future medicine. This study provides an overview of the current knowledge on the therapeutic potential of MSCs and MSC‐derived extracelullar vesicles, as well as methods for improving their biological activities to promote angiogenesis and tissue repair.

## 1 INTRODUCTION

**FIGURE 1:**
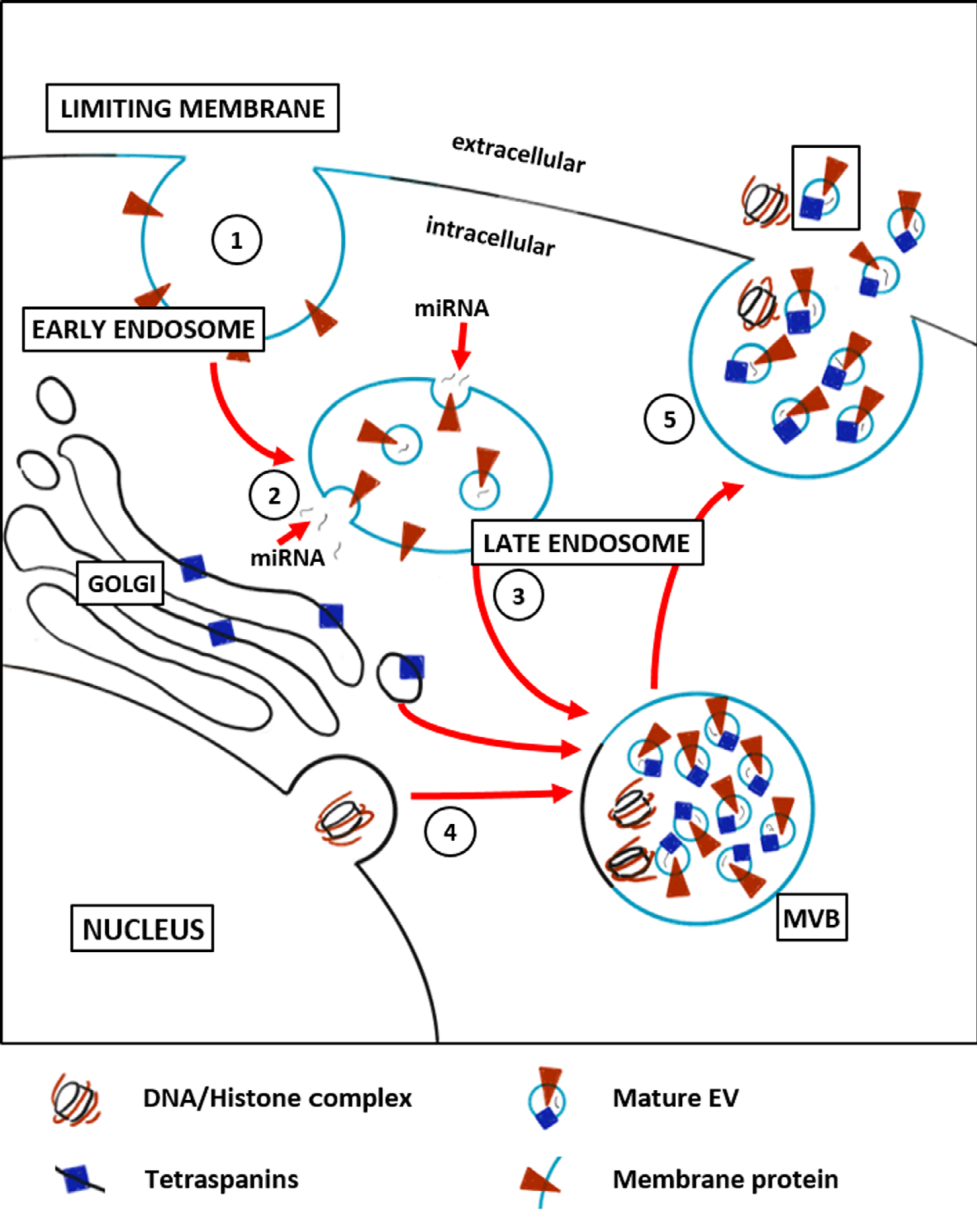
Exosome biosynthesis. (1) Early endosomes are formed by inward budding of the limiting membrane of cells. Surface proteins (orange triangles) may be incorporated into the early endosomal membrane. (2) Early endosomes undergo a maturation process to form late endosomes, in which the biogenesis of exosomes occurs by continuous invagination of the limiting membrane. (3) This particular type of late endosome, which ends up accumulating numerous small intraluminal vesicles with a diameter of 40 to 150 nm is called multivesicular body (MVB). During this process, cytosolic components (eg, miRNAs) are actively packed into the vesicles. In addition, communication with the Golgi apparatus through bidirectional vesicle exchange leads to the incorporation of tetraspanins (blue rectangles) into the membrane of the vesicles. (4) Besides that, cytosolic histone‐bound DNA fragments can be transported to MVBs via the autophagosome pathway. (5) Finally, MVBs either fuse with the plasma membrane causing the release of their content into the extracellular environment, or fuse with lysosomes for degradation of their cargo.

In both developed and developing countries, cardiovascular disease (CVD) is a major cause of morbidity and mortality,^1^ and most importantly, ischemic heart disease such as acute myocardial infarction (MI) is a leading cause of heart failure. While obstruction to blood flow can be effectively treated by common surgical and catheter‐based interventions, achieving cures for microvascular disease remains an elusive goal. The concept of promoting the perfusion of ischemic tissue through angiogenesis has been considered as a highly promising treatment strategy for CVD. Previous attempts to induce neocapillarization in ischemic tissue involved the targeted delivery of various proangiogenic growth factors and nucleic acids encoding them, as well as physical interventions to stimulate angiogenic processes.^2^ However, as none of them proved to be sufficiently effective to reverse end‐organ ischemia and prevent loss‐of‐function, other strategies had to be pursued. With the advent of cell therapies for nonhematological disorders in the 1990s, the idea of using viable cells to ameliorate or reverse tissue ischemia has rapidly gained traction.^3^ Given their ease of isolation, robustness in culture, multilineage differentiation potential in vitro, and partially restricted immunogenicity,^4^ mesenchymal stromal cells (MSCs) have been proposed as a promising tool for translational research in cardiology. In recent years, much work has been done to improve the functional properties of MSCs in terms of cell retention and survival of grafted cells, and to elicit their proangiogenic effects. For example, it has been hypothesized that the microenvironment of injured tissue is not conducive for cell engraftment and retention, and that the paracrine effect of transplanted MSCs lasts for only 24 to 48 hours.^5^ To overcome these limitations, various scaffolds for cell transplantation were tested and showed promising results for the use in cardiac applications.^6^, ^7^ MSC transplantation to repair damage caused by MI and restore cardiac function has been demonstrated in both animal experiments and patients.^8^, ^9^, ^10^, ^11^ However, recent meta‐analyses failed to show consistent improvement in infarct size or left ventricular function.^12^, ^13^ Consequently, the initial assumption that transplanted stem or progenitor cells support neovascularization by differentiation into endothelial cells was soon replaced by the notion of their predominantly paracrine function by producing and secreting small molecules responsible for proangiogenic effects, such as cytokines, chemokines, and growth factors.^14^ Besides releasing a variety of soluble factors, MSCs have been shown to secrete extracellular vesicles (EVs) that are important mediators of cell‐to‐cell communication.^15^ Among the known subtypes of EVs, endosome‐derived exosomes carrying proteins, metabolites, lipids, and various RNAs have emerged as physiologically relevant components of the MSC secretome^16^ (Figure 1). Earlier reports demonstrated that the paracrine activity of the MSC secretome has a therapeutic effect on a wide range of diseases and tissue injury in myocardium, kidney, liver, and lung.^17^, ^18^, ^19^, ^20^ The elucidation of paracrine effects thus not only improves our understanding of vascular pathologies, but also enhances the ability to facilitate neocapillarization (ie, endothelial sprouting) for regeneration purposes. In this article, we summarize ways to stimulate angiogenesis with the help of MSCs and their derived EVs, thereby enhancing tissue repair in a variety of pathologies associated with insufficient angiogenesis. We also present the latest advances in the identification of regulatory microRNAs (miRNAs) encapsulated in EVs and discuss their role in promoting angiogenesis.

## 2 ROLE OF MSCs IN ANGIOGENESIS

The human body contains approximately 90 000 km of blood vessels that supply all cells and tissues with vital nutrients and oxygen needed for survival and proliferation.^21^ The stimulation of new blood capillary vessel formation through the process of angiogenesis is an integral part of tissue growth and repair. It has been hypothesized that MSCs are part of the perivascular niche in various organs and play an important role in the orchestration of neocapillarization,^22^, ^23^ which has rapidly attracted considerable interest in the scientific community. In addition, due to their in vitro multipotent differentiation potential into mesenchymal lineages, including osteoblasts, chondrocytes, myocytes, and adipocytes, the idea was raised that they could also replenish lost tissue in vivo.^24^ The therapeutic rationale for MSC treatment, for example, for acute MI patients, is to repair damaged heart tissue by cardiomyocyte differentiation and to provide growth factors to induce angiogenesis, to stimulate resident cardiac stem cell migration and commitment to cardiomyocytes. Most evidence suggests that the beneficial effects of MSCs are mainly caused by the secretion of a variety of bioactive paracrine factors.^25^ Especially for bone marrow‐derived MSCs, numerous small molecules have been demonstrated to induce angiogenesis both in vitro and in vivo; key factors are summarized in Table 1.

**TABLE 1:** Key proangiogenic factors secreted by MSCs

Short name	Long name	Reference
ANG	Angiogenin	26
ANGPT1	Angiopoietin‐1	27
EGF	Epidermal growth factor	28
FGF‐2	Fibroblast growth factor‐2	29
G‐CSF	Granulocyte‐colony stimulating factor	30
HGF	Hepatocyte growth factor	31
IL‐6	Interleukin‐6	32
IL‐8	Interleukin‐8	33
MCP‐1	Monocyte chemotactic protein‐1	34
PDGF	Platelet‐derived growth factor	35
PlGF	Placental growth factor	36
SDF‐1	Stromal cell‐derived factor‐1	37
TGF‐alpha	Transforming growth factor alpha	38
TGF‐beta	Transforming growth factor beta	39
TNF‐alpha	Tumor necrosis factor alpha	39
VEGF	Vascular endothelial growth factor	39

Abbreviation: MSCs, mesenchymal stromal cells.

Vascular endothelial growth factor (VEGF) and fibroblast growth factor‐2 (FGF‐2) are two of the most studied factors that regulate angiogenesis.^39^ Given that elevated levels can induce cell proliferation and migration of endothelial cells, coordinated regulation of VEGF and FGF‐2 expression is required to elicit the proangiogenic effects of MSCs. Another interesting proangiogenic protein is tumor necrosis factor alpha (TNF‐alpha), as its effect on angiogenesis depends on the concentration and the duration of treatment. Therefore, it might have a dual role in angiogenesis: high doses of TNF‐alpha were found to inhibit angiogenesis in mice in vivo, while low doses promoted it.^40^ In addition to classical angiogenic factors, MSCs also secret EVs that carry a variety of biomolecules capable of regulating angiogenesis both in vitro and in vivo.^41^, ^42^ EVs were proposed as key agents in the modulation of angiogenesis^43^ and have been shown to improve angiogenesis in a number of studies, including mouse and rat models of burn injuries, skin wounds, acute kidney injury, acute MI, and limb ischemia.^44^, ^45^, ^46^

## 3 ENHANCEMENT OF THE ANGIOGENIC POTENTIAL OF MSCs

MSCs can be obtained from a variety of tissues, such as bone marrow, adipose tissue, and umbilical cord tissue, with bone marrow being the most common stem cell source.^47^ Efforts to maximize the secretion of proangiogenic factors by MSCs are expected to substantially increase the beneficial role of MSCs in regenerative medicine. Several studies have shown that preconditioning of MSCs by hypoxia enhances the proangiogenic effects of MSCs,^48^, ^49^ which might be a valuable strategy for boosting their clinical potential and therapeutic efficacy upon transplantation. Exposure of MSCs to reduced oxygen partial pressure induces the expression of genes involved in migration and homing, mainly regulated by hypoxia‐inducible factor‐1 alpha (HIF‐1 alpha).^50^ HIF‐1 alpha is constitutively expressed in most cell types, including cardiac cells. Under normoxic conditions, it is inactive due to ubiquitin‐mediated proteasomal degradation and transcriptional inhibition.^51^ However, under hypoxic conditions, HIF‐1 alpha becomes rapidly stabilized and its accumulation results in higher gene expression of proangiogenic factors, such as VEGF and transforming growth factor beta,^52^, ^53^ as well as increased release of EVs from MSCs.^54^ Overexpression of HIF‐1 alpha also promotes incorporation of Jagged1, a Notch ligand that increases angiogenesis, into MSC‐derived EVs, suggesting that an active HIF‐1 alpha phenotype can be transmitted to surrounding cells.^55^ In addition, hypoxia‐preconditioned MSCs show a higher cell viability, enhanced proliferation potential, decreased production of reactive oxygen species, increased antioxidant glutathione production, and higher superoxide dismutase levels.^56^ Other stress conditions that may be of interest for enhancing the angiogenic potential of MSCs include pH variation and calorie restriction.^57^ However, given that modification of culture conditions is a rather indirect process for increasing the angiogenic activity of MSCs, as it affects not only one specific molecule but many factors, it may in turn lead to serious side effects. Apart from altering the overall culture environment, several growth factors have been shown to enhance the regenerative capacity of MSCs in vitro. For instance, pretreatment of MSCs with epidermal growth factor or transforming growth factor alpha increased the release of proangiogenic factors such as VEGF and hepatocyte growth factor, which play a central role in inducing angiogenesis and improving oxygen supply to ischemic tissues.^58^, ^59^ In addition, it has been shown that MSCs, when cultured on collagen‐coated patches, are less fibrogenic and secrete more cardiotrophic factors.^60^ Besides modulating culture conditions or using additives, the genetic modification of MSCs was also investigated.^61^ Although MSCs naturally possess an enormous inherent therapeutic potential, gene therapy is being used to modify MSCs to further enhance their efficacy and even extend the range of diseases for which MSCs could be applied. MSCs can be easily transduced by clinically available viral vector systems, including retrovirus and lentivirus.^62^ This technique leads to efficient production of angiogenic factors and, because viral vectors can be integrated into the host genome, to long‐term gene expression.^63^ Numerous animal studies have reported the success of genetically engineered MSCs as a gene delivery vehicle. For example, Xu et al^64^ have used a lentiviral vector to generate MSCs that overexpress angiopoietin‐1, a proangiogenic protein that induces endothelial survival and vascular stabilization. Another study by Song et al^65^ showed that the introduction of v‐myc into human MSCs using a lentiviral gene delivery system resulted in increased MSC secretion of VEGF and thus increased vessel formation. However, since applications of these vectors elicited adverse side effects including toxicities, immuno‐ and oncogenicity,^66^ many clinical studies using viral vectors were terminated. Therefore, nonviral vectors have been continuously studied and have become an attractive alternative for MSC modification.^67^ As one example, Bandara et al^68^ described a novel nonviral minicircle vector to deliver the endothelial nitric oxide synthase (eNOS) transgene to MSCs. Overexpression of eNOS has been shown to improve the ability of MSCs to treat ischemic heart damage following coronary artery occlusion. In a rat model of acute MI, the authors demonstrated that transplantation of eNOS‐overexpressing MSCs significantly reduced MI size, increased capillary density, and corrected hemodynamic parameters. In addition, in recent years, transfection of MSCs by modified mRNAs has gained considerable traction as a promising strategy to prime MSCs for targeted delivery of therapeutic molecules at a controlled rate.^69^ Another approach to multiply the therapeutic potential of systemically applied MSCs is to module their homing and interaction with target cells by surface coating. For example, Chou et al^70^ showed that bone marrow‐derived MSCs transfected with 1,3‐fucosyltransferase VI, an enzyme transforming native CD44 on MSCs into a hematopoietic cell E‐/L‐selectin ligand, increased homing to injured endothelial cells. Similarly, Zou et al^71^ coated mouse adipose tissue‐derived MSCs (AMSCs) with antibodies to kidney injury molecule‐1, a protein that is upregulated in damaged kidneys, and injected them into mice with renal artery stenosis. These AMSCs showed selective homing compared to untreated AMSCs, leading to improved renal perfusion and capillary density as well as attenuation of oxidative damage and fibrosis. Besides improving the homing efficiency to and retention of MSCs in a target tissue, enhancing MSC survival is a major milestone in improving the effectiveness of MSC‐based therapy.^72^ Strategies like preconditioning with hyperoxia or repeated episodes of short‐term exposure to hypoxia have been found to promote the viability and proliferation of MSCs.^73^ In addition, studies have provided evidence that stromal cell‐derived factor‐1 alpha (SDF‐1 alpha) can suppress apoptosis in MSCs and promote cardiomyocyte survival. Tang et al^74^ found that 1 week after cell implantation, the number of SDF‐1 alpha‐modified MSCs was five times higher than that of wild‐type MSCs in a rat model of MI. Nevertheless, more studies are needed to further improve the survival of MSCs after transplantation in heart tissue.

To date, it has not been conclusively investigated whether MSCs transplantation and systemic application can promote or cause neoplasia and possibly cancer.^75^ Studies have shown that due to their perivascular origin, MSCs can differentiate into pericytes or endothelial cells, which ultimately supports tumor vascularization and growth. However, the data on the interaction of MSCs with different tumor types are ambiguous.^76^ There is evidence in the literature to support the hypothesis that MSCs can inhibit capillarization in vitro in a dose‐dependent manner.^77^ Furthermore, the group of Otsu et al^77^ showed tumor recession in vivo after coinoculation of melanoma cells with MSCs. Most clinical trials using MSCs for myocardial regeneration screened their subjects for tumor formation after MSCs injections. In clinical safety studies with unmodified MSCs for myocardial regeneration, no neoplasms associated with MSC application were observed.^78^ Nevertheless, since proangiogenic modifications of MSCs carry the risk of promoting existing tumor growth, it is important to carefully examine patients for pre‐existing neoplasms.

## 4 MSC‐DERIVED EVs AS AN ALTERNATIVE TO MSC TRANSPLANTATION

MSCs could be used in an autologous setting to exclude immune responses of the recipient and thereby preserve their regenerative properties.^79^ However, autologous MSC applications have some limitations, including availability and decreased biological activity when isolated from elderly donors and patients with systemic diseases. For example, MSCs isolated from older patients showed a reduction in superoxide dismutase activity and an increase in reactive oxygen species, resulting in oxidative damage in MSCs and, consequently, apoptosis and senescence.^80^ In addition, autologous MSC extraction and in vitro expansion prior to implantation is time‐consuming, making it difficult to use them to treat acute diseases such as MI. These shortcomings, coupled with the evidence that MSCs have immunomodulatory properties and are less immunogenic compared to other cell types^14^ have stimulated the development of allogeneic MSC products obtained from young and healthy donors. Given their anti‐inflammatory and immune‐evasive mechanisms, off‐the‐shelf allogeneic MSCs that can be administered immediately were considered as a promising option for tissue repair. However, in clinical trials, the overall therapeutic effect was limited, similar to autologous MSCs.^12^ Additionally, the use of viable cells still carries inherent risks such as microvasculature obstruction, immune rejection, and proarrhythmic side effects. EVs derived from MSCs can overcome many of these concerns associated with the use of living cells, while having therapeutic effects similar to those achievable by the originating MSCs themselves.^81^ In conclusion, rather than transplanting exogenous MSCs, MSC‐derived EVs, even from allogeneic sources, offer a great alternative because they are nonproliferative, less immunogenic, and easier to store and deliver than MSCs.^82^ However, as a prerequisite for application, it must be ensured that MSC‐derived EVs can be produced in sufficient quantity and quality and that they are able to effectively mediate pro‐regenerative and immunomodulatory effects of the parental cells.

## 5 PROANGIOGENIC CHARACTERISTICS OF MSC‐DERIVED EVs

EVs, such as exosomes, are small secretory vesicles carrying a large number of bioactive molecules, including proteins, metabolites, lipids, and RNAs.^83^ Besides other and larger types of EVs, they are produced by most cell types under normal and pathophysiological conditions and serve as messengers of the intercellular network, allowing the exchange of cellular components between cells.^42^ In detail, classical exosomes are generated in multivesicular bodies and excreted in the extracellular environment when these compartments fuse with the plasma membrane^84^ (Figure 1). They can then either be taken up by target cells localized in the microenvironment or transported to distant sites via biological fluids. Upon arrival at the target cells, exosomes can deliver their content directly into the cytoplasm of the target cell or may be surrounded by the plasma membrane and be disintegrated in the cytoplasm, where their content is released.^85^ For recognition and internalization by the target cells, exosomes have specific proteins on their surface, such as tetraspanins.^42^ In regenerative medicine, EVs secreted by MSCs can stimulate proliferation and inhibit apoptosis of recipient cells. Accordingly, their proangiogenic effects are related to their ability to sustain the viability and proliferation of endothelial cells.^86^ However, studies have also reported that MSC‐derived EVs are potent regulators of tumorigenesis. For example, Zhu et al^87^ showed an increase in tumor incidence and growth when human gastric and colon cancer cell lines were mixed with MSC‐derived EVs and then injected subcutaneously into mice. Similarly, Ren et al^88^ have shown, using a xenograft model, that intravenous injection of hypoxia‐conditioned MSC‐EVs significantly increases tumor development. It is therefore critical to identify which molecules transferred by EVs induce cancer pathways and which tumor types can benefit from MSC‐EV treatment.

Several studies have shown that MSC‐derived EVs contain cytokines and growth factors, and accumulating evidence indicates that angiogenesis can also be specifically regulated by different encapsulated RNAs, including miRNAs.^89^ MiRNA is a class of highly conserved, single‐stranded, 19 to 22 nucleotide long, noncoding small RNAs that regulate gene expression at the post‐transcriptional level by targeting 3′‐untranslated regions of specific mRNAs.^90^ Upon binding, miRNAs inhibit mRNA translation or cause mRNA degradation, thus suppressing protein synthesis. Although more than 2000 miRNAs are present in humans^91^ and nearly 800 miRNAs have been identified in the human heart at this time,^92^ the nature of target transcripts is unknown for many of them. Meanwhile, only a few miRNAs have been described to promote angiogenesis; Table 2 shows a selection of known miRNAs with proangiogenic activity.

**TABLE 2:** Selection of miRNAs with proangiogenic properties

miRNA	Regulated targets (selection)	Reference
miR‐let‐7	ALK5, FASLG, TSP‐2	93, 94, 95
miR‐9^a^	ECAD, SOCS5	96, 97, 98
miR‐10a^a^	KLF4, PTEN	99, 100
miR‐10b^a^	HOXD10, KLF4, SDC1	101, 102
miRNA‐17~92^a^	CTGF, TSP‐1	103
miR‐21^a^	CHIP, PDCD4, PTEN, SMAD7, SPRY1, STAT3	104, 105, 106, 107, 108
miR‐23a^a^	PHD1, PHD2, TSGA10, ZO‐1	109, 110
miR‐26b	COX2, CTGF, OCT4, SMAD1	111, 112, 113
miR‐27b	DLL4, SPRY2	114, 115
miR‐30b	DLL4, JDP2	116, 117
miR‐30d^a^	MYPT1	118
miR‐31	FIH‐1	119
miR‐93^a^	ITGB8	120
miR‐125a	DLL‐4	121
miR‐126	PIK3R2, SPRED1	122, 123, 124
miR‐130a^a^	GAX, HOXA5, RUNX3, TFPI2	125, 126, 127
miR‐132^a^	p120RasGAP	128
miR‐135b^a^	FIH‐1, LATS2	129, 130
miR‐145	TMOD3	131
miR‐146a^a^	BRCA1, NF2, PAK1, RAC1	132, 133
miR‐150^a^	c‐Myb, SRCIN1, TP53	134, 135, 136, 137, 138, 139
miR‐155^a^	VHL	140
miR‐181a^a^	SRCIN1	141
miR‐181b^a^	GATA6, PDCD10,	142
miR‐182^a^	BRCA1, FOXO3, HMGA2, MITF‐M, MTSS1	143
miR‐194^a^	TSP‐1	144
miR‐210^a^	EFNA3	145, 146
miR‐214	ATM	147
miR‐217	FOXO3A, KRAS, SIRT1	148, 149
miR‐296^a^	HGS	150
miR‐378^a^	FUS‐1, SUFU	151
miR‐382^a^	PTEN	152
miR‐424	CUL2	153
miR‐433	DKK1	154
miR‐467^a^	TSP‐1	155
miR‐494^a^	CASP2	156
miR‐1246^a^	PML	157

Abbreviations: ALK5, activin receptor‐like kinase 5; ATM, ataxia telangiectasia mutated protein; BRCA1, breast cancer protein 1; CASP2, caspase‐2; CHIP, carboxyl terminus of the heat‐shock cognate 70‐interacting protein; COX2, cyclooxygenase‐2; CTGF, connective tissue growth factor; CUL2, cullin 2; DKK1, dickkopf Wnt signaling pathway inhibitor 1; DLL4, delta‐like ligand 4; ECAD, e‐cadherin; FASLG, Fas ligand; FIH‐1, factor‐inhibiting hypoxia‐inducible factor 1; FOXO3, forkhead‐box‐protein O3; GATA6, GATA‐binding factor 6; GAX, growth arrest‐specific homeobox; HGS, hepatocyte growth factor‐regulated tyrosine kinase substrate; HMGA2, high‐mobility group AT‐hook 2; HOXA5, homeobox A5; HOXD10, homeobox D10; ITGB8, integrin B8; JDP2, jun dimerization protein 2; KLF4, Krüppel‐like factor 4; LATS2, large tumor suppressor kinase 2; MITF‐M, microphthalmia‐associated transcription factor type M; MTSS‐1, metastasis suppressor‐1; MYPT1, myosin phosphatase targeting subunit 1; NF2, neurofibromin 2; PAK1, p21‐activated kinase 1; PDCD4, programmed cell death protein 4; PDCD10, programmed cell death protein 10; PHD1, prolyl hydroxylase 1; PHD2, prolyl hydroxylase 2; PIK3R2, phosphoinositide‐3‐kinase regulatory subunit 2; PML, promyelocytic leukemia protein; PTEN, phosphatase and tensin homolog; RAC1, Ras‐related C3 botulinum toxin substrate 1; p120RasGAP, Ras GTPase‐activating protein 1; RUNX3, Runt‐related transcription factor 3; SDC1, syndecan‐1; SOCS5, suppressor of cytokine signaling 5; SPRED1, sprouty‐related EVH1 domain containing 1; SPRY1, sprouty homologue 1; SPRY2, sprouty homologue 2; SRCIN1, SRC kinase signaling inhibitor 1; STAT3, signal transducer and activator of transcription 3; SUFU, suppressor of fused; TFPI2, tissue factor pathway inhibitor 2; TMOD3, tropomodulin 3; TP53, tumor protein p53; TSP‐1, thrombospondin‐1; TSP‐2, thrombospondin‐2; VHL, von Hippel‐Lindau tumor suppressor; ZO‐1, zonula occludens‐1.

^a^ miRNAs that have been shown to also play a role in promoting angiogenesis in tumors.

For instance, with regard to miRNAs incorporated into MSC‐derived EVs, miRNA‐21 activates the protein kinase B/extracellular signal‐regulated kinase signaling pathway leading to the overproduction of VEGF.^104^ MiR‐126 exerts its activity by targeting phosphoinositide‐3‐kinase regulatory subunit 2 and sprouty‐related EVH1 domain containing 1, two negative regulators of VEGF signaling.^122^ MiR‐130a is a strong positive regulator of angiogenesis because it targets, for example, the antiangiogenic factors growth arrest‐specific homeobox and homeobox A5.^125^ MiR‐135b and miR‐31 contribute to angiogenesis by accelerating HIF‐1 alpha transcriptional activity via inhibition of factor‐inhibiting hypoxia‐inducible factor 1, an asparaginyl hydroxylase enzyme that suppresses HIF‐1 alpha.^119^, ^129^ Likewise, miR‐23a directly targets prolyl hydroxylase 1 and 2, leading to HIF‐1 alpha stabilization.^109^ However, despite the benefits of selective miRNAs for regenerative medicine approaches by inducing angiogenesis, there is a close relationship between vascularity and tumor expansion.^158^ For example, the miRNA‐17~92 cluster has been shown to increase angiogenesis both in vitro and in vivo, and its predominance was observed in a variety of human cancers.^159^ Similarly, plasma miRNA‐21 levels have been described as a marker for various types of tumors, such as breast, colon, prostate, ovarian, pancreatic, and lung cancer.^160^ In addition, since miRNAs do not perfectly complement their target mRNAs, they may target multiple genes whose protein products act on different signaling pathways and thus dysregulate several networks in tumor cells.^161^ Given their pivotal role in carcinogenesis, off‐target effects of miRNAs should be well characterized before evaluating their use in clinical settings. Taken together, although aberrant angiogenesis may contribute to pathological conditions, including growth and dissemination of tumors, miRNA application is more welcome for the induction of angiogenesis than its repression.^162^, ^163^

## 6 EVs AS VEHICLES FOR THE TARGETED DELIVERY OF PROANGIOGENIC MOLECULES

An EV‐based delivery system for proangiogenic factors offers great benefits such as low toxicity, low immunogenicity, high blood circulation stability, biocompatibility, and biological barrier permeability. Apart from the fact that stress situations, such as hypoxia, can alter the composition of EVs, the loading of EVs with proangiogenic factors is a more specific approach to facilitate angiogenesis. To date, various methods have been proposed for loading EVs which can be classified into either cargo loading during formation or after isolation. One promising approach for cargo loading during EV formation is the transfection of MSCs with DNA encoding therapeutically relevant compounds, which are then released into secreted EVs. However, because overexpression of a particular factor does not ensure increased presence in EVs, loading of EVs after release with proangiogenic factors or the vectors encoding them was considered. For instance, by applying an electric field to a suspension of EVs and the therapeutic cargo, pores are created in the membrane, thereby facilitating movement of the cargo into the lumen of EVs.^164^ Besides the efficacy of EVs, methods for optimal delivery to the heart are still being investigated. Previous studies have used both intracoronary and intramyocardial injections, with the latter being more effective. For example, Gallet et al^165^ showed that EVs from human cardiosphere‐derived cells administered to pigs in both acute and chronic models of cardiac ischemia lead to reduced infarct size and preserved systolic function after intramyocardial but not intracoronary delivery. Whether these improvements will be maintained in the long‐term remains to be investigated. However, although intramyocardial injections are acceptable in animal studies, this method is not clinically appealing because of its invasive nature. Ideally, EVs should be administered intravenously. Nonetheless, a challenge with the use of exogenously administered EVs is that they may be nonspecifically trapped in nontargeted organs, particularly in the lungs and liver, resulting in insufficient targeting of myocardial ischemia.^166^ As with cells, attempts to modify EVs as effective tools directly targeting ischemic myocardium have been considered. One method is to restructure transmembrane proteins of EVs to fuse them with ligands or homing peptides, thereby conferring the ability of EVs for targeting tissues bearing the corresponding receptors. Recently, a new peptide sequence, CSTSMLKAC, has been discovered that can preferentially target the ischemic region of the heart, resulting in increased specificity and efficiency of EVs targeting the ischemic myocardium.^166^ Another major challenge to the clinical application of EVs is that a high dose is required to improve angiogenesis to a physiologically relevant extent.^167^ Although methods for isolating EVs are continuously being developed and optimized, the typical yield of an EV isolation can be less than 1 μg of total EV protein from 1 mL of culture medium,^168^ while the therapeutic dose of EVs is normally in the range of 10 to 100 μg of protein in mouse models.^169^ In turn, the effective dose in humans could be an order of magnitude or more to compensate for the rapid clearance of EVs from the body. In sum, EVs are promising carriers for proangiogenic molecules, and future efforts should investigate their specific delivery to target organs and the optimal dose. Other important issues to be addressed are the precise mechanism of action of exogenously administered EVs in vivo, the appropriate time window for EV administration, and the route of administration that achieves maximum efficacy without side effects.^170^, ^171^

## 7 CONCLUSIONS

MSCs have been explored as a versatile and widely used cell source in regenerative medicine and tissue engineering. Owing to their proangiogenic potential, which is mainly mediated by paracrine factors, they are a promising treatment strategy for diseases caused by insufficient angiogenesis such as MI. But instead of transplanting autologous or allogenic MSCs, a new option is cell‐free therapy, where MSCs are first cultivated and their EVs are then isolated and further manipulated to achieve a more proangiogenic cargo. Evidence supports the superiority of this approach over stromal cell transplantation. Although there are still some drawbacks with respect to the production of sufficiently large amounts of EVs, sample impurities and the inability to produce EVs without the use of cells, EVs constitute a major focus of proangiogenic therapy. Proteins, mRNAs, and miRNAs contained in EVs can be transferred to recipient cells in order to induce their reprogramming to promote angiogenesis. Interestingly, encapsulated miRNAs have recently emerged as key positive regulators of angiogenesis in pathological settings of insufficient blood supply and thus represent promising new tools for CVD treatment. However, to manifest this potential, several challenges for miRNA therapeutics need to be addressed, including the efficiency of the delivery system and the currently incomplete understanding of their biology, as some miRNAs also play a role in promoting angiogenesis in tumors. Future mechanistic studies with EVs should carefully monitor potential off‐target and dose‐dependent effects.

In conclusion, cell‐free MSC‐derived EVs loaded with proangiogenic factors represent a feasible option in situations of insufficient angiogenesis, such as acute MI and ischemia. Consequently, EVs may constitute a promising platform for noncellular regenerative therapies to complement or even replace the use of MSCs in tissue regeneration and repair.

## CONFLICT OF INTEREST

V.F. declared consultant/advisory role with Novartis Pharma, Berlin Heart, Biotronik, Edwards, Medtronic, grant funding from Abbott, Medtronic, Boston Scientific, JOTEC, and reimbursements from Edwards, JOTEC, Abbott, Medtronic. The other authors declared no potential conflicts of interest.

## AUTHOR CONTRIBUTIONS

T.N.S., S.N.: conceptualized and wrote the article and performed the literature search; A.G.D., E.B., Z.X.: supported the literature search; M.S., C.S., V.F.: supported with writing and proof‐reading the article.

## Data Availability

Data sharing is not applicable to this article as no new data were created or analyzed in this study.
